# Albumin Reduces Paracentesis-Induced Circulatory Dysfunction and Reduces Death and Renal Impairment among Patients with Cirrhosis and Infection: A Systematic Review and Meta-Analysis

**DOI:** 10.1155/2013/295153

**Published:** 2013-10-08

**Authors:** Chun Shing Kwok, Lukasz Krupa, Ash Mahtani, Duncan Kaye, Simon M. Rushbrook, Martin G. Phillips, William Gelson

**Affiliations:** ^1^Norfolk and Norwich University Hospital, Colney Lane, Norwich NR4 7UY, UK; ^2^Norwich Medical School, University of East Anglia, Norwich NR4 7TJ, UK

## Abstract

*Background.* Studies have suggested that albumin has a value in cirrhotic patients undergoing paracentesis but its value in infection and sepsis is less clear. We planned to perform a meta-analysis of the risk of adverse outcomes in cirrhotic patients with and without albumin use. *Methods.* We searched MEDLINE and EMBASE in January 2013 for randomized studies of cirrhotic patients that reported the risk of adverse events and mortality with albumin and no albumin exposure. We performed random effects meta-analysis and assessed heterogeneity using the *I^2^* statistic. *Results.* Our review included 16 studies covering 1,518 patients. The use of albumin in paracentesis was associated with significantly reduced risk of paracentesis-induced circulatory dysfunction (OR 0.26 95%, CI 0.08–0.93) and there was a nonsignificant difference in death, encephalopathy, hyponatraemia, readmission, and renal impairment. Compared to the other volume expanders, albumin use showed no difference in clinical outcomes. In cirrhotic patients with any infection, there was a significant reduction in mortality (OR 0.46 95%, CI 0.25–0.86) and renal impairment (OR 0.34 95%, CI 0.15–0.75) when albumin was used. *Conclusion.* The use of albumin in cirrhotic patients is valuable in patients with any infection and it reduces the risk of circulatory dysfunction among patients undergoing paracentesis.

## 1. Introduction

Intravenous administration of human albumin solution is frequently used in patients with decompensated liver cirrhosis during the drainage of ascites. In the absence of intravenous albumin, postparacentesis circulatory dysfunction occurs in approximately 70% of patients [1, 2] and is associated with increased mortality because of hepatorenal syndrome and dilutional hyponatraemia [3, 4]. In addition, albumin administration is recommended in patients with spontaneous bacterial peritonitis (SBP) [5, 6].

Albumin is a plasma expander that increases cardiac preload and peripheral vascular resistance, attenuates endothelial dysfunction, reduces renal failure, and improves survival [7, 8]. As a result, albumin is widely accepted as a therapy in large volume paracentesis and spontaneous bacterial peritonitis, but the evidence supporting this therapy in other settings is less clear.

Bacterial infections are common in patients with advanced cirrhosis and are a major cause of hospital admissions and mortality [9–12]. Approximately 30–40% of cirrhotic patients with severe infection develop renal failure and this is a poor prognostic factor [13–16]. The infection that most often causes renal failure is spontaneous bacterial peritonitis, followed by urinary tract infection and cellulitis.

Few data exist regarding the effect of albumin administration in patients with non-SBP infections. The question of whether albumin infusion also improves outcome in patients with bacterial infections other than SBP remains unanswered. Studies to date failed to answer this question due to small sample size [17] and further research should be performed to answer this question.

In this systematic review, we provide a comprehensive and up-to-date overview of the existing evidence regarding the use of albumin in cirrhotic patients. We were specifically interested in three questions. Is albumin beneficial in cirrhotic patients undergoing large volume paracentesis? Is albumin useful in cirrhotic patients with infections, with particular focus on SBP and non-SBP infections? Is albumin superior to other volume expanders in cirrhotic patients?

## 2. Methods

### 2.1. Eligibility Criteria

We selected randomized controlled trials (RCTs) that evaluated the use of human serum albumin in patients with cirrhosis. The specific inclusion criteria for the studies were (1) parallel group randomized trial with albumin in one of the treatment arms for any duration of therapy; (2) placebo or alternative control arm; (3) clear reporting of adverse events. The adverse events of interest were death, encephalopathy, hyponatraemia, paracentesis-induced circulatory dysfunction, readmission, renal impairment, gastrointestinal bleeding, infection resolution, and sepsis/severe infection. There was no restriction on cirrhotic patients and those with tense ascites requiring paracentesis, and those with infections including SBP were included.

### 2.2. Search Strategy

We searched MEDLINE and EMBASE through OvidSP in January 2013 using the Haynes optimized search strategy (Health Information Research Unit, McMaster University) (see Supplementary Material 1 available online at http://dx.doi.org/10.1155/2013/295153) [18]. We also checked the bibliographies of the included trials and recent review articles for relevant studies.

### 2.3. Study Selection and Data Extraction

One reviewer (Chun Shing Kwok) and one of two other reviewers (Ash Mahtani or Duncan Kaye) independently scanned all titles and abstracts for studies that met the inclusion criteria and excluded any articles that clearly did not fulfil the selection criteria. Full reports of potentially relevant trials and studies were retrieved and independently checked by two reviewers (Lukasz Krupa and William Gelson). We then independently collected information on study design, drug exposure, study location, characteristics of participants, and relevant outcomes onto a spreadsheet (Chun Shing Kwok, Ash Mahtani, and Duncan Kaye). Where there was any uncertainty or discrepancies, the article was discussed between the reviewers (Lukasz Krupa, Simon M. Rushbrook, Martin G. Phillips, and William Gelson) to determine if the studies should be included. We also contacted authors if there were any areas that required clarification.

### 2.4. Risk of Bias Assessment

We evaluated the quality of studies in accordance with the recommendations of the Cochrane Handbook and this assessment included sequence generation, allocation concealment, blinding of participant, personnel and outcome assessors, incomplete outcome data, selective reporting, and baseline differences in participants [32].

We aimed to generate funnel plots to assess the possibility of publication bias, provided that there were >10 studies available in the meta-analysis, with no evidence of substantial statistical heterogeneity.

### 2.5. Data Analysis

We used RevMan 5.022 (Nordic Cochrane Centre) to conduct random effects meta-analysis using pooled odds ratios (OR). We chose to pool raw outcome data to yield unadjusted risk ratios (which may be particularly susceptible to confounding). In view of the potential diversity of study participants and interventions, we chose to perform sensitivity analysis based on groupings:

1. cirrhotic patients receiving paracentesis,
2. cirrhotic patients receiving albumin compared to other volume expanders,
3. cirrhotic patients with infections, SBP, and non-SBP infections.

### 2.6. Statistical Heterogeneity

Statistical heterogeneity was assessed using *I* ^2^ statistic, with *I* ^2^ values of 30–60% representing a moderate level of heterogeneity [33].

## 3. Results

**Figure 1:**
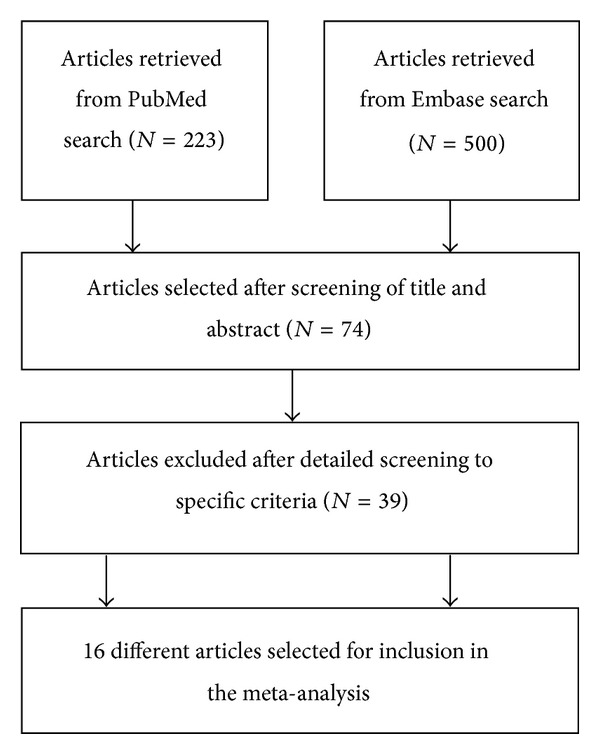
Flow diagram of the process of article selection for meta-analysis.

The search results yielded 16 relevant RCTs with 1,518 patients from Egypt, France, Korea, Argentina, Mexico, Spain, USA, Italy, and China. Four studies [2, 21, 23, 30] evaluated albumin versus no albumin/saline, and eight studies [19, 20, 22, 24, 25, 29, 34, 35] compared plasma expander to albumin. Four other studies [26, 28, 31] of cirrhotic patients with infection compared the use of antibiotics with and without albumin. Study durations varied from 5 days to 27 months. The process of selection is shown in Figure 1 and the main characteristics of the included studies are described in Table 1. The outcomes, interventions, and quality assessments of the included studies are shown in Table 2 and Supplementary Material 2.

For methodological quality assessment, the majority of trials (10 trials) had adequate sequence generation for randomization but only two trials had adequate allocation concealment (Supplementary Material 2). Blinding of patients, personnel, and outcome assessors was unclear in the majority of studies and most studies did not have any evidence of selective reporting and baseline difference.

### 3.1. Is Albumin Useful in Paracentesis?

Three studies were included in the analysis of whether albumin was useful in paracentesis [2, 23, 30]. The use of albumin was associated with significant reduction in paracentesis-induced circulatory dysfunction (OR 0.26 95% CI 0.08–0.93). No significant difference was observed for the risk of any other outcomes with and without albumin use. There was a nonsignificant trend towards reduction in hyponatraemia, paracentesis-induced circulatory dysfunction, readmission, and renal impairment.

### 3.2. Is Albumin Better Than Other Volume Expanders in Paracentesis?

Eight studies evaluated the use of volume expanders compared to albumin [19, 20, 22, 24, 25, 29, 34, 35]. None of these studies compared the use of albumin versus other volume expanders outside of the paracentesis context. The use of albumin resulted in no significant advantage in risk of death, encephalopathy, hyponatraemia, gastrointestinal bleeding, readmission, renal impairment, and sepsis/severe infection.

### 3.3. Is Albumin Useful in Infections/Sepsis?

**Figure 2:**
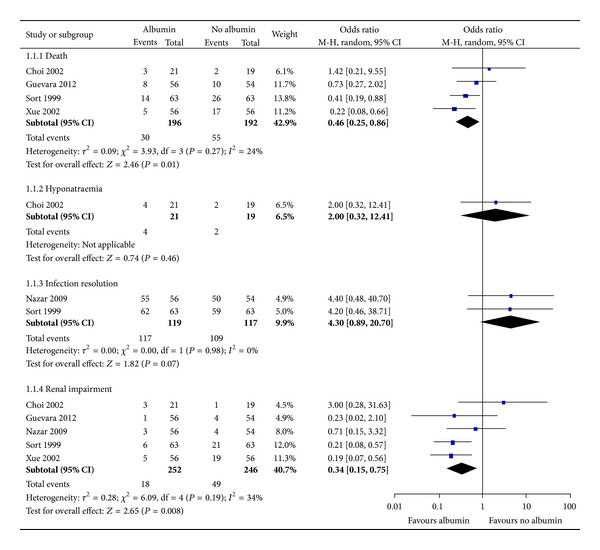
Albumin versus no albumin among cirrhotic patients with infections.

Five studies were included in the analysis of albumin use in sepsis. Two studies [26, 28] included participants with non-SBP infection while three studies included participants with SBP [8, 21, 31]. In the context of any infection, the use of albumin was associated with reduced risk of death OR 0.46 95% CI 0.25–0.86, *I* ^2^ = 24% and renal impairment OR 0.34 95% CI 0.15–0.75, *I* ^2^ = 34%. There was a nonsignificant trend towards infection resolution and increased risk of hyponatraemia. Subgroup analysis considering SBP showed that the albumin use was associated with reduced risk of death. No significant difference was observed for non-SBP infection (Table 3). The pooled results of the risk evaluations are shown in Figure 2 and Table 2.

## 4. Discussion

Our results suggest that albumin has a value in the treatment of cirrhotic patients in the contexts of large volume paracentesis and infections. There is no significant advantage of albumin compared to other plasma expanders for paracentesis. In paracentesis, albumin reduces the risk of paracentesis-induced circulatory dysfunction. In cases of cirrhotic patients with infections, death and renal impairment can be reduced with the use of albumin. Therefore, cirrhotic patients at high risk of circulatory dysfunction during paracentesis should receive albumin or an alternative plasma expander, and cirrhotic patients with sepsis or infection at high risk of renal impairment or death should receive albumin.

Broadly, our findings support the AASLD and EASL recommendations for the management of large volume paracentesis [5, 6]. The use of other plasma expanders is not supported as there is insufficient evidence according to the guidelines. While albumin may cost more than plasma expanders, the use of albumin is justified as there is evidence that there are fewer 30-day liver related events among the patients treated with albumin [27]. Both guidelines support the use of albumin in spontaneous bacterial peritonitis [5, 6] but there are currently no guidelines for non-SBP infections.

There are a number of potential explanations for our findings. Human albumin is a major plasma protein and acts as an intravascular volume expander. It is produced in the liver and its concentration is reduced with hepatic dysfunction. It is responsible for 80% of the colloid osmotic pressure of plasma; therefore, the intravenous administration of albumin is associated with a rapid increase in the circulating blood volume. In the context of infection and sepsis, the acute inflammatory response has a vasodilatory effect. This leads to circulatory collapse which is compounded by a lack of albumin to maintain oncotic pressure in the intravascular compartment. In addition, it has other physiological functions such as transport of water insoluble endogenous and exogenous substances such as anti-inflammatory mediators, hormones, and antibiotics in sepsis. It also acts as a circulatory antioxidant which may prevent cellular injury from reactive oxygen species in sepsis and ischaemia.

Our review builds on the finding of the meta-analysis by Bernardi et al. [36]. Similar to their findings, we found that the use of albumin reduces the risk of paracentesis-induced circulatory dysfunction. However, we did not find that there was any significant difference when albumin was compared to other volume expanders. Their study was limited to patients receiving paracentesis while our study also considers patients with infection. 

Our study has several advantages. All the studies included were prospective randomized trials. We were able to consider cirrhotic patients in several different settings including those requiring paracentesis with tense ascites as well as those with SBP and non-SBP infection. In addition, we were able to consider a variety of outcomes including death, encephalopathy, hyponatraemia, paracentesis-induced circulatory dysfunction, readmission renal impairment, gastrointestinal bleed, infection resolution, and sepsis/severe infection.

There are several limitations in this review. The quality of the studies is generally poor as blinding was not used. Furthermore, the sample size of the studies is small and only a few studies were included in each pooled analysis. The duration of followup was also variable among the studies.

We would recommend three studies of albumin and plasma expanders which are in the context of paracentesis, non-SBP, and SBP infections. In addition to clinical outcomes such as need for death, encephalopathy, hyponatraemia, GI bleeding, readmission, renal impairment, sepsis, and need for liver transplant, this study should further consider the cost implications. The most important of the possible studies is that of albumin versus plasma expanders in infections (non-SBP and SBP) as these questions have not been answered. In addition, a study of different doses of albumin could help determine the dosing regimen that would lead to the best clinical outcomes. We believe that the ideal trial would be an adequately powered multicentre double-blinded trial that has followup of at least 1 month and corrects for liver disease severity.

## 5. Conclusions

In conclusion, the findings of this meta-analysis support the use of albumin for preventing paracentesis-induced circulatory collapse and reducing the risk of death and renal failure in cirrhotic patients with infections limited to SBP. More studies are needed to evaluate if albumin reduces adverse outcomes among cirrhotic patients without paracentesis or infections. There is no evidence to support the use of albumin over other plasma expanders for paracentesis.

## Supplementary Material

The Supplementary Material contains the search strategy, quality assessment of included studies and Prisma statement.

## Figures and Tables

**Table 1:** Study design, patient characteristics and selection criteria.

Study	Design; year of study; country	Number of participants	Mean age	Male %	Selection criteria
Abdel-Khalek and Arif 2010 [19]	Randomized trial; Apr 2008 to Feb 2009; Egypt.	135; 68 albumin; 67 hydroxyethyl starch.	47	79	Patients with cirrhosis and tense ascites which were unresponsive to low-sodium diet and diuretic therapy.

Altman et al. 1998 [20]	Randomized trial; unclear dates; France.	65; 33 albumin; 27 hydroxyethyl starch.	56	73	Patients with cirrhosis and tense ascites.

Choi et al. 2002 [21]	Randomized trial; Jun 1998 to Feb 2001; Korea.	40; 21 albumin; 19 no albumin.	56	78	Patients with cirrhosis and spontaneous bacterial peritonitis.

Fassio et al. 1992 [22]	Randomized trial; Apr 1988 to Mar 1990; Argentina.	40; albumin 21; dextran-70 20.	54	76	Patients with cirrhosis and tense ascites.

Garcia-Compean et al. 1993 [23]	Randomized trial; unclear dates; Mexico.	35; 17 albumin; 18 no albumin.	56	58	Patients with cirrhosis and tense ascites.

García-Compean et al. 2002 [24]	Randomized trial; unclear dates; France.	69; albumin 48; dextran-40 48.	59	82	Patients with cirrhosis and tense ascites.

Gines et al. 1988 [2]	Randomized trial; Jul 1985 to Dec 1986, Spain.	105; 52 albumin; 53 no albumin.	57	66	Patients with cirrhosis and tense ascites.

Gines et al. 1996 [25]	Randomized trial; unclear dates, Spain.	289; albumin 97; dextran-70 93; polygeline 99.	58	70	Patients with cirrhosis and tense ascites.

Guevara et al. 2012 [26]	Randomized trial; unclear dates; Spain.	110; 56 albumin and antibiotics; 54 antibiotics.	56	64	Patients with cirrhosis and non-spontaneous bacterial peritonitis infections.

Moreau et al. 2006 [27]	Randomized pilot study; May 2000–Jun 2001; France.	68; 30 albumin; 38 polygeline.	55	82	Patients with cirrhosis and ascites who needed to receive a plasma expander because of paracentesis, renal impairment or marked hyponatraemia.

Nazar et al. 2009 [28]	Randomized trial; unclear dates; Spain.	110; 56 albumin and antibiotics; 54 antibiotics.	NA	NA	Patients with cirrhosis and non-SBP infections.

Planas et al. 1990 [29]	Randomized trial; USA.	88; 43 albumin; 45 dextran-70.	59	63	Patients with cirrhosis and tense ascites treated with paracentesis.

Salemo 1991 [35]	Randomized trial; May 1986–July 1989; Italy.	54; 27 albumin; 27 hemaccel.	54	87	Patients with cirrhosis and refractory ascites.

Sola-Vera et al. 2003 [30]	Randomized trial; Jan 1997–Dec 2000; Spain.	72; 37 albumin; 35 saline.	61	65	Patients with cirrhosis and tense ascites.

Sort et al. 1999 [8]	Randomized trial; Nov 1995–Sept 1997; Spain.	126; 63 cefotaxime and albumin; 63 cefotaxime.	61	64	Patients with cirrhosis and spontaneous bacterial peritonitis.

Xue et al. 2002 [31]	Randomized trial; unclear dates; China.	112; 56 albumin and ceftriaxone; 56 ceftriaxone.	NA	NA	Patients with cirrhosis, ascites and spontaneous bacterial peritonitis.

NA: not available.

**Table 2:** Treatment groups, followup, and results.

Study	Albumin group	Control group	Duration of followup	Results
Abdel-Khalek and Arif 2010 [19]	Paracentesis and IV 20% albumin 8 g/L of ascitic fluid removed.	Paracentesis and hydroxyethyl starch 6%.	Up to 11 months.	Bacterial infection 3/68 versus 3/67, death 5/68 versus 6/67, encephalopathy 3/68 versus 2/67, GI bleeding 5/68 versus 5/67, readmissions 32/68 versus 37/67.

Altman et al. 1998 [20]	Paracentesis and IV 20% albumin 100 mL if <2 L of ascites removed, 200 mL if 2–5 L of ascitic fluid removed.	Paracentesis and hydroxyethyl starch 200 mL/L of ascetics fluid removed.	Up to 15 days after paracentesis.	Encephalopathy 1/33 versus 0/27, GI bleeding 0/33 versus 1/27, hyponatraemia 0/33 versus 1/27, severe infection 2/33 versus 3/27.

Choi et al. 2002 [21]	Paracentesis and albumin 6–8 g/L of ascitic fluid removed.	General management.	Followup at 7 days.	Death 3/21 versus 2/19, encephalopathy 2/21 versus 0/19, hyponatremia 4/21 versus 2/19, renal impairment 3/21 versus 1/19.

Fassio et al. 1992 [22]	Paracentesis with albumin 6 g/L of ascitic fluid removed.	Paracentesis with dextran 70 6 g/L of ascitic fluid removed.	In-hospital outcomes.	Death 6/21 versus 6/20, encephalopathy 1/21 versus 2/20, GI bleeding 3/21 versus 0/20, hyponatremia 4/21 versus 3/20, readmission 6/21 versus 5/20, renal impairment 1/21 versus 1/20, sepsis 2/21 versus 1/20.

Garcia-Compean et al. 1993 [23]	Paracentesis with albumin 5 g/L of ascitic fluid removed.	Paracentesis without albumin.	5 days.	Encephalopathy 1/17 versus 0/18, hyponatremia 1/17 versus 1/18, renal impairment 1/17 versus 2/18.

García-Compean et al. 2002 [24]	Paracentesis with albumin infusion 8 g/L of ascitic fluid removed.	Paracentesis with 8 g/L Dextran solution (10 mg Dextran-40 and 5 g sorbitol per 100 mL)	14 months.	Death 11/48 versus 18/48, hyponatremia 3/48 versus 5/48, readmission 30/48 versus 34/48, renal impairment 7/48 versus 2/48.

Gines et al. 1988 [2]	Paracentesis 4–6 L/day until resolution and IV 20% albumin infusion (40 g after each tap).	Paracentesis without albumin.	27 months.	Death 18/51 versus 14/50, hyponatremia 1/51 versus 2/52, readmission 29/51 versus 36/50, renal impairment 0/51 versus 1/50.

Gines et al. 1996 [25]	Paracentesis with IV albumin 8 g/L of ascitic fluid removed.	Group II: Paracentesis and IV dextran-70 (8 g/L of ascitic fluid) and Group 3: Paracentesis with IV polygeline (8 g/L of ascitic fluid).	NA	Bacterial infection 5/97 versus 3/99, deaths 2/97 versus 6/99, encephalopathy 3/97 versus 5/99, GI bleeding 1/97 versus 1/99, hyponatremia 14/97 versus 19/99, PPCD 17/92 versus 37/98, renal impairment 7/97 versus 10/99.

Guevara et al. 2012 [26]	Antibiotics plus 20% albumin (1.5 g/kg then 1 g/kg).	Best infection care with antibiotics but not albumin.	3 months.	Death 8/56 versus 10/54, renal impairment 1/56 versus 4/54.

Moreau et al. 2006 [27]	Paracentesis and 20% albumin replacement.	Paracentesis and 3.5% polygeline.	6 months.	Hyponatraemia 8/29 versus 15/38.

Nazar et al. 2009 [28]	Antibiotics plus albumin (1.5 g/kg then 1 g/kg).	Best infection care with antibiotics but not albumin.	3 months.	Infection resolution 55/56 versus 50/54, renal impairment 3/56 versus 4/54.

Planas et al. 1990 [29]	Paracentesis and IV 20% albumin 8 g/L of ascitic fluid removed.	Paracentesis and dextran-70 8 g/L ascitic fluid removed.	27.5 weeks versus 23.7 weeks.	Death 13/43 versus 17/45, encephalopathy 3/43 versus 3/45, GI bleeding 1/43 versus 3/45, hyponatraemia 3/43 versus 4/45, readmissions 24/43 versus 29/43, renal impairment 1/43 versus 1/45, severe infection 1/43 versus 2/45.

Salemo 1991 [35]	Paracentesis and IV 20% albumin 30 mL/L of ascitic fluid removed.	Paracentesis and IV haemaccel 3.5% 150 mL/L of ascitic fluid removed.	NA.	Encephalopathy 2/27 versus 2/27, hyponatraemia 4/27 versus 5/27, readmissions 17/27 versus 12/27, renal impairment 1/27 versus 1/27.

Sola-Vera et al. 2003 [30]	Paracentesis and IV 20% albumin 8 g/L of ascitic fluid removed.	Paracentesis and 170 mL of 3.5% saline/L of ascites removed.	NA.	Death 1/37 versus 1/35, hyponatraemia 2/37 versus 5/35, PICD 4/37 versus 11/35, renal impairment 2/37 versus 3/35.

Sort et al. 1999 [8]	IV cefotaxime 2 g dosing based on serum creatinine and 20% albumin 1.5 g/kg during first 6 hours, then 1 g/kg on day 3.	IV cefotaxime dosing based on creatinine.	NA.	Death in hospital 6/63 versus 18/63, death at three months 14/63 versus 26/63, infection resolution 62/63 versus 59/63, renal impairment 6/63 versus 21/63.

Xue et al. 2002 [31]	IV ceftriaxone plus IV albumin 0.5–1.0 g/kg within 6 hours of enrolment, then same amount on 3rd day and every 3 days thereafter.	IV ceftriaxone 3 g/day adjusted based on serum creatinine.	NA.	Renal impairment 5/56 versus 19/56, deaths 5/56 versus 17/56.

NA: not available.

**Table 3:** Summary of results.

Outcome or subgroup	Studies	Participants	Odds ratio (95% confidence interval)
*Is albumin useful in paracentesis? *			
1.1.1 Death	2	173	1.36 (0.61, 3.04)
1.1.2 Encephalopathy	1	35	3.36 (0.13, 88.39)
1.1.3 Hyponatraemia	3	210	0.47 (0.13, 1.66)
1.1.4 Paracentesis-induced circulatory dysfunction	1	72	0.26 (0.08, 0.93)
1.1.5 Readmission	1	101	0.51 (0.22, 1.18)
1.1.6 Renal impairment	3	208	0.51 (0.13, 1.98)

*Is albumin better than other volume expander in paracentesis? *			
1.2.1 Death	5	556	0.63 (0.38, 1.03)
1.2.2 Encephalopathy	6	574	0.87 (0.42, 1.80)
1.2.3 Hyponatraemia	7	602	0.70 (0.44, 1.12)
1.2.4 Gastrointestinal bleeding	5	520	0.90 (0.35, 2.31)
1.2.5 Readmission	5	412	0.83 (0.56, 1.24)
1.2.6 Renal impairment	5	475	1.09 (0.51, 2.34)
1.2.7 Sepsis/severe infection	3	189	0.74 (0.21, 2.62)

*Is albumin useful in infections overall? *			
1.3.1 Death	4	388	0.46 (0.25, 0.86)
1.3.2 Hyponatraemia	1	40	2.00 (0.32, 12.41)
1.3.3 Infection resolution	2	236	4.30 (0.89, 20.70)
1.3.4 Renal impairment	5	498	0.34 (0.15, 0.75)

*Is albumin useful in spontaneous bacterial peritonitis? *			
1.4.1 Death	3	278	0.39 (0.18, 0.84)
1.4.2 Infection resolution	1	126	4.20 (0.46, 38.71)
1.4.3 Renal impairment	3	278	0.32 (0.10, 1.04)

*Is albumin useful in non-spontaneous bacterial peritonitis infections? *			
1.5.1 Death	1	110	0.73 (0.27, 2.02)
1.5.2 Infection resolution	1	110	4.40 (0.48, 40.70)
1.5.3 Renal impairment	1	110	0.23 (0.02, 2.10)
