# Supplementary material for: Albumin Reduces Paracentesis-Induced Circulatory Dysfunction and Reduces Death and Renal Impairment among Patients with Cirrhosis and Infection: A Systematic Review and Meta-Analysis
Source: Biomed Res Int. 2013 Oct 8;2013:295153. doi: 10.1155/2013/295153 (PMC3816020; doi:10.1155/2013/295153)
Supplement: Supplementary file 1 — The Supplementary Material contains the search strategy, quality assessment of included studies and Prisma statement. [file 295153.f1.pdf]

**Appendix 1:** Search Strategy

1 randomized controlled trial.pt. or randomized.mp. or placebo.mp.
2 cirrhosis.mp.
3 albumin.mp.
4 1 and 2 and 3

## Appendix 2: Quality assessment of included studies

| Studies | Sequence Generation | Allocation concealment | Blinding of participants and personnel | Blinding of outcome assessors | Incomplete outcome data | Selective outcome reporting | Baseline differences in participants |
|---|---|---|---|---|---|---|---|
| Abdel-Khalek 2010 | Adequate, based on random numbers generated by SAS V6.12 statistical software | Unclear, allocation concealment not stated | Unclear, not stated | Unclear, not stated | Unclear, not stated | Adequate, no evidence of selective reporting. | Adequate, no baseline variation |
| Altman 1998 | Adequate, random number table | Unclear, not stated | Unclear, not stated | Unclear, not stated | Adequate | Adequate, no evidence of selective reporting. | Adequate, no significant baseline variation |
| Choi 2002 | Unclear | Unclear | Unclear | Unclear | Unclear | Unclear | Adequate, no baseline variation |
| Fassio 1992 | Unclear, doesn't state method of randomization | Unclear, not stated | Unclear, not stated | Unclear, not stated | Adequate | Adequate, no evidence of selective reporting. | Adequate, no baseline variation |
| Garcia-Compean 1993 | Adequate, randomized with random number table | Unclear | Unclear, not stated | Unclear, not stated | Adequate | Adequate, no evidence of selective reporting. | Adequate, no significant baseline variation |
| Garcia-Compean 2002 | Adequate, random number table | Unclear, not stated | Unclear, not stated | Unclear, not stated | Adequate | Adequate, no evidence of selective reporting. | Adequate, no baseline variation |
| Gines 1988 | Adequate, randomly allocated to two groups (random number table) | Adequate, randomization was independent in each hospital | Unclear, not stated | Unclear, not stated | Adequate | Adequate, no evidence of selective reporting. | Adequate, no baseline variation |
| Gines 1996 | Unclear, doesn't state method of randomization | Unclear, not stated | Unclear, not stated | Unclear, not stated | Adequate | Adequate, no evidence of selective reporting. | Adequate, no baseline variation |
| Guevara 2012 | Adequate, sealed envelopes | Unclear, allocation concealment not stated | Unblinded | Inadequate, unblinded | Adequate | Adequate, no evidence of selective reporting. | Adequate, no baseline variation |

| | | | | | | | |
|---|---|---|---|---|---|---|---|
| Moreau 2006 | Adequate, based on random numbers generated by SAS V6.12 statistical software | Unclear, allocation concealment not stated | Adequate, only nurses were aware | Unclear, not stated | Adequate | Adequate, no evidence of selective reporting. | Adequate, no baseline variation |
| Nazar 2009 | Unclear, doesn't state method of randomization | Unclear, allocation concealment not stated | Unclear, not stated | Unclear, not stated | Unclear, not stated | Unclear | Inadequate, not stated |
| Planas 1990 | Adequate, randomly allocated to two groups (random number table) | Adequate, randomization was independent in each hospital | Unclear, not stated | Unclear, not stated | Adequate | Adequate, no evidence of selective reporting. | Adequate, no baseline variation |
| Salemo 1991 | Unclear | Unclear | Unclear | Unclear | Unclear | Adequate, no evidence of selective reporting. | Adequate, no significant baseline variation |
| Sola-Vera 2003 | Adequate, random number table and sealed envelopes | Unclear, not stated | Unclear, not stated | Unclear, not stated | Unclear, not stated | Adequate, no evidence of selective reporting. | Adequate, no baseline variation |
| Sort 1999 | Adequate, sealed envelopes containing the numbers of treatment assignments based on random numbers generated by the SAS module | Unclear, not stated | Unclear, not stated | Adequate, investigators blinded | Unclear, not stated | Adequate, no evidence of selective reporting. | Adequate, no baseline variation apart from in WCC and ascitic fluid PMN count |
| Xue 2002 | Unclear, not stated | Unclear, not stated | Unclear, not stated | Unclear, not stated | Unclear, not stated | Unclear, very little analysis | Unclear, not stated |

## PRISMA Statement

| Section/topic | # | Checklist item | Reported on page # |
|---|---|---|---|
| **TITLE** | | | |
| Title | 1 | Identify the report as a systematic review, meta-analysis, or both. | 1 |
| **ABSTRACT** | | | |
| Structured summary | 2 | Provide a structured summary including, as applicable: background; objectives; data sources; study eligibility criteria, participants, and interventions; study appraisal and synthesis methods; results; limitations; conclusions and implications of key findings; systematic review registration number. | 2 |
| **INTRODUCTION** | | | |
| Rationale | 3 | Describe the rationale for the review in the context of what is already known. | 3-4 |
| Objectives | 4 | Provide an explicit statement of questions being addressed with reference to participants, interventions, comparisons, outcomes, and study design (PICOS). | 3-4 |
| **METHODS** | | | |
| Protocol and registration | 5 | Indicate if a review protocol exists, if and where it can be accessed (e.g., Web address), and, if available, provide registration information including registration number. | NA |
| Eligibility criteria | 6 | Specify study characteristics (e.g., PICOS, length of follow-up) and report characteristics (e.g., years considered, language, publication status) used as criteria for eligibility, giving rationale. | 5 |
| Information sources | 7 | Describe all information sources (e.g., databases with dates of coverage, contact with study authors to identify additional studies) in the search and date last searched. | 5 |
| Search | 8 | Present full electronic search strategy for at least one database, including any limits used, such that it could be repeated. | Appendix |
| Study selection | 9 | State the process for selecting studies (i.e., screening, eligibility, included in systematic review, and, if applicable, included in the meta-analysis). | 5-6 |
| Data collection process | 10 | Describe method of data extraction from reports (e.g., piloted forms, independently, in duplicate) and any processes for obtaining and confirming data from investigators. | 5-6 |
| Data items | 11 | List and define all variables for which data were sought (e.g., PICOS, funding sources) and any assumptions and simplifications made. | 5-7 |

| | | | |
|---|---|---|---|
| Risk of bias in individual studies | 12 | Describe methods used for assessing risk of bias of individual studies (including specification of whether this was done at the study or outcome level), and how this information is to be used in any data synthesis. | 6 |
| Summary measures | 13 | State the principal summary measures (e.g., risk ratio, difference in means). | 5-7 |
| Synthesis of results | 14 | Describe the methods of handling data and combining results of studies, if done, including measures of consistency (e.g., $I^2$) for each meta-analysis. | 6-7 |

| Section/topic | # | Checklist item | Reported on page # |
|---|---|---|---|
| Risk of bias across studies | 15 | Specify any assessment of risk of bias that may affect the cumulative evidence (e.g., publication bias, selective reporting within studies). | 6 |
| Additional analyses | 16 | Describe methods of additional analyses (e.g., sensitivity or subgroup analyses, meta-regression), if done, indicating which were pre-specified. | 6-7 |
| **RESULTS** | | | |
| Study selection | 17 | Give numbers of studies screened, assessed for eligibility, and included in the review, with reasons for exclusions at each stage, ideally with a flow diagram. | 8-10, Figure |
| Study characteristics | 18 | For each study, present characteristics for which data were extracted (e.g., study size, PICOS, follow-up period) and provide the citations. | 8-10, Table |
| Risk of bias within studies | 19 | Present data on risk of bias of each study and, if available, any outcome level assessment (see item 12). | 8, Appendix |
| Results of individual studies | 20 | For all outcomes considered (benefits or harms), present, for each study: (a) simple summary data for each intervention group (b) effect estimates and confidence intervals, ideally with a forest plot. | Table |
| Synthesis of results | 21 | Present results of each meta-analysis done, including confidence intervals and measures of consistency. | 8-10 |
| Risk of bias across studies | 22 | Present results of any assessment of risk of bias across studies (see Item 15). | 8, Appendix |
| Additional analysis | 23 | Give results of additional analyses, if done (e.g., sensitivity or subgroup analyses, meta-regression [see Item 16]). | 8-10 |
| **DISCUSSION** | | | |
| Summary of evidence | 24 | Summarize the main findings including the strength of evidence for each main outcome; consider their relevance to key groups (e.g., healthcare providers, users, and policy makers). | 11 |

| | | | |
|---|---|---|---|
| Limitations | 25 | Discuss limitations at study and outcome level (e.g., risk of bias), and at review-level (e.g., incomplete retrieval of identified research, reporting bias). | 13 |
| Conclusions | 26 | Provide a general interpretation of the results in the context of other evidence, and implications for future research. | 13 |
| **FUNDING** | | | |
| Funding | 27 | Describe sources of funding for the systematic review and other support (e.g., supply of data); role of funders for the systematic review. | 1 |
